# Context matters—the phased development of an adaptable food literacy intervention: Up for Cooking

**DOI:** 10.1093/heapro/daad071

**Published:** 2023-07-14

**Authors:** Lisa S E Harms, Patricia van Assema, Jessica S Gubbels, Sanne M P L Gerards, Evelyne Linssen, Lieve Vonken, Kathelijne M H H Bessems

**Affiliations:** Department of Health Promotion, School of Nutrition and Translational Research in Metabolism, Maastricht University, PO Box 616, 6200 MD Maastricht, The Netherlands; Department of Health Promotion, School of Nutrition and Translational Research in Metabolism, Maastricht University, PO Box 616, 6200 MD Maastricht, The Netherlands; Department of Health Promotion, School of Nutrition and Translational Research in Metabolism, Maastricht University, PO Box 616, 6200 MD Maastricht, The Netherlands; Department of Health Promotion, School of Nutrition and Translational Research in Metabolism, Maastricht University, PO Box 616, 6200 MD Maastricht, The Netherlands; Department of Knowledge & Innovation, Public Health Service South Limburg, PO Box 33, 6400 AA Heerlen, The Netherlands; Department of Health Promotion, School of Care and Public Health Research Institute, Maastricht University, PO Box 616, 6200 MD Maastricht, The Netherlands; Department of Health Promotion, School of Nutrition and Translational Research in Metabolism, Maastricht University, PO Box 616, 6200 MD Maastricht, The Netherlands

**Keywords:** adaptation, context, intervention development, implementation

## Abstract

Adapting interventions to the context increases the impact and sustainability of interventions. Literature acknowledges the need to adapt existing interventions and that these adaptations should be clearly reported. However, little is known about *how to* incorporate adaptation from the beginning. This paper argues that interventions should be developed and adaptations should be made using an ongoing non-linear approach. An action-oriented research approach with feedback loops is proposed. We illustrate this with the development of a food literacy intervention ‘Up for Cooking’ (Dutch: Zin in Koken) and present lessons learned in developing, implementing and studying such adaptable interventions. Interventions should clearly define and differentiate the intervention function and form. Implementers, in turn, should be encouraged to tailor interventions within a form that fits with a specific context. Sufficient time, continuous adaptation based on co-creation, feedback loops and interdisciplinary collaboration are important prerequisites for the development of adaptable interventions.

**Contribution to Health Promotion**

- Interventions should fit with the context, taking into account the perspective of target groups, other stakeholders and external circumstances.
- We propose a non-linear approach to developing adaptable interventions, differentiating the function and the form of the intervention.
- We recommend understand the context you are working in; embrace collaborations; learn from your mistakes; incorporate flexibility and consider guidance for practice.
- These recommendations could help researchers and health promotion professionals to develop adaptable interventions.

## BACKGROUND

### Fidelity, fit and adaptable interventions

A systematically planned intervention development process increases intervention reach, implementation and impact (Allen *et al.*, 2017). The core of this process is selecting theoretical methods to address relevant determinants, and translating these methods into strategies or intervention components, such as workshops or newsletters (Eldredge *et al.*, 2016). Although, fidelity is considered important for successful implementation and intervention effectiveness, its tension with adaptation of interventions has been central to the debate for years (Dusenbury *et al.*, 2003; Elliott and Mihalic, 2004; Bopp *et al.*, 2013; Escoffery *et al.*, 2018). Research on the need to adapt interventions to new contexts is developing rapidly (Cohen *et al.*, 2008; Moore *et al.*, 2021). Today, researchers acknowledge that it is not the extent to which intervention components are fully and precisely implemented that is crucial, but the fit between the intervention and the context (Cohen *et al.*, 2008; Chambers and Norton, 2016; Moore *et al.*, 2021). This *intervention-context fit* improves adequate implementation (Dusenbury *et al.*, 2003; Castro *et al.*, 2004; Cohen *et al.*, 2008; Chambers and Norton, 2016) and is even expected to promote intervention sustainment (Scheirer and Dearing, 2011; Chambers *et al.*, 2013).

Despite these efforts to emphasize the importance of intervention-context fit and adaptable interventions, the tension with fidelity remains. Interestingly, most adaptation literature focusses on the adaptation of previously developed evidence-based interventions (Moore *et al.*, 2021). However, we believe that adaptation should be considered from intervention development onwards (Castro *et al.*, 2004; Hawe *et al.*, 2004; Brand *et al.*, 2018), but research on approaches to develop such adaptable interventions is limited. In this position paper, we propose a non-linear and action-oriented research approach to adaptable intervention development and implementation. Our paper starts with a brief summary of the literature, including terminology and definitions. It then describes the steps of a non-linear approach based on the development of Up for Cooking (UfC): an adaptable cooking intervention to promote food literacy skills among Dutch adults or families with a lower socioeconomic status (SES). At each step, input (i.e. context, stakeholders and action-oriented research approach) and output (i.e. intervention function and form, and implementation strategies) are described in detail. Our paper concludes with implications for researchers and health promotion professionals regarding the development and implementation of adaptable interventions.

### Differentiating function, form and context

Researchers have stressed that intervention developers should differentiate between intervention function and form (Hawe *et al.*, 2004; Hawe, 2015; Perez Jolles *et al.*, 2019; Lengnick-Hall *et al.*, 2021). *Function* represents the core characteristics or components that play a role in the behaviour change process and therefore define the purpose of an intervention (Hawe *et al.*, 2004; Lengnick-Hall *et al.*, 2021). The absence of these components would compromise the fidelity and effectiveness of the intervention (Hawe, 2015; Perez Jolles *et al.*, 2019). *Form* reflects how an intervention is implemented (e.g. methods, components, point of emphasis), and allows for tailoring to the context (Hawe *et al.*, 2004). The absence of a proposed form can be replaced or can lead to an evolution to another form and thus does not compromise intervention fidelity (Hawe *et al.*, 2004; Perez Jolles *et al.*, 2019). This implies that rather than standardizing specific behaviour change methods into core intervention components (e.g. a workshop or newsletter) or intervention delivery (e.g. session duration), it is the underlying function that should be standardized (Castro *et al.*, 2004; Hawe *et al.*, 2004; Hawe, 2015). For example, in the case of a cooking intervention for food literacy, the steps of planning, selecting, preparing and eating a healthy meal are core components of food literacy (Vidgen and Gallegos, 2014), defining the intervention function. These core components are applicable in interventions for different contexts, in different forms. The tailoring of the form should be based on several aspects of the *context*. First, the characteristics of *the target group* must be taken into account to achieve impact (Castro *et al.*, 2004; Durlak and Dupre, 2008). A cooking intervention should match participants’ characteristics, such as literacy level or financial resources, and personal objectives such as motivation to consume healthier meals. Second, implementation is preceded by the adoption of the intervention local stakeholders, and should align with the objectives and interests of the *organizations and stakeholders* adopting the intervention. For example, the objective of a primary school may be to focus on parenting practices in the cooking intervention, whereas community centers may want to focus on the social participation of hard-to-reach groups. Finally, *external circumstances* (e.g. for this project COVID-19) may influence the implementation conditions and require different cooking intervention forms (e.g. on-site to online delivery).

### Alternative approaches to intervention development

Embracing the need for intervention-context fit corresponds to the notion of complex systems, where interventions are seen as events within a system (Hawe *et al.*, 2009; Hawe, 2015). Embracing this complexity requires changes in intervention development and related research. This includes a shift from traditional intervention development, which often largely follows a linear approach (i.e. development, effectiveness, implementation and evaluation) to an ongoing, non-linear approach that allows for continuous adjustments during new implementations (May *et al.*, 2016; Rutter *et al.*, 2017). One way to do this is to use an action-oriented research approach with feedback loops, where the ‘output’ of research is continuously fed back into the system as an ‘input’ for practice. We propose to fully exploit the benefits of action-oriented research by creating a continuous process of adaptation from the start. Only then can we achieve an optimal fit between the intervention, the target group and the context. This paper describes the phased non-linear development of UfC to demonstrate the application of an action-oriented research approach for the development of adaptable interventions.

## DEVELOPMENT OF UFC

The development process of UfC (2019–2022) embraced the previously described views on adaptation and intervention-context fit, using a non-linear approach with feedback loops. As input to this process, we distinguish: (i) the context in which development and implementation take place; (ii) the stakeholders involved in the co-creation and implementation of the intervention and (iii) the action-oriented research that guided the development. Output is: (i) function and form of the intervention and (iis) implementation strategies. Table 1 provides an overview of each phase of the development.

**Table 1:** Phased non-linear development of UfC, detailing input and output in each phase

*Phase 1: Initial intervention development (January–December 2019)*		
Input	Context	Professionals and policy makers aimed to identify and engage vulnerable adults in nutrition education (*needs of adopting organizations and stakeholders*). Start of the development, implementation and evaluation of a cooking intervention for parents with low literacy (*the target group*) in community centers.
	Stakeholders	- Health promotion researchers with expertise in the development, implementation and evaluation of interventions; - Regional Municipal Health Services (*project leader*), cook with affinity for social activities and healthy eating (*course leader*); - Local and regional professionals with ample experience in working with vulnerable groups; - Community centers at which the intervention could take place; - Vulnerable target groups: adults with a lower SES and/or lower literacy skills.
	Action-oriented research	- Theoretical and empirical evidence on nutrition education; - Input of professionals in the field through interim bilateral contact and co-creation; - Pilot testing supported by process evaluation (i.e. observations, interviews).
Output	Intervention function and form	The initial intervention aimed to empower participants to cook a healthy meal. The initial form was a four-session on-site cooking intervention with room to choose points of emphasis (e.g. healthy eating, literacy skills, saving money on groceries or parenting practices).
	Implementation strategies	Recruitment strategy and materials, and a first draft of the CL manual.
*Phase 2: Broadening the target group and improving intervention adaptability (January–June 2020)*		
Input	Context	Libraries and social organizations reached out to adopt UfC as a method to reach and engage parents with a lower SES (*new target group*) to address healthy eating, social cohesion and literacy (*new needs adopting organizations and stakeholders*).
	Stakeholders	- Health promotion researchers, PL and CL (*project team*); - Hosts from a social non-profit restaurant addressing poverty and social cohesion through UfC; - Low literacy manager positioned at a local library aiming to reach low-literate parents through UfC.
	Action-oriented research	- Pilot testing supported by process evaluation (i.e. observations, interviews); - Co-creation with the project team.
Output	Intervention function and form	Clear distinction between *intervention function* (i.e. promoting food literacy skills) and *intervention forms* (i.e. literacy, social cohesion, healthy eating, parenting and budget).
	Implementation strategies	Updated CL manual and website for the first step of the scale-up.
*Phase 3: Adaptation to the online setting (September 2020–July 2022)*		
Input	Context	Closure of community centers and primary schools due to COVID-19 measures (*external circumstances*). Demand to adapt the intervention in order to continue implementation (*adopting organizations and stakeholders*) as well as interest to reach families at home (*target group*).
	Stakeholders	- Researchers, PL and CL (project team); - Low literacy manager positioned at a local library aimed to reach low-literate parents through UfC; - Regional branch of the Dutch initiative *Jong Leren Eten* involved in childhood food education, willing to adopt and disseminate UfC further among primary schools; - Management and teachers of nine primary schools willing to adopt UfC, recruiting children and parents.
	Action-oriented research	- Theoretical and empirical evidence on online cooking interventions; - Co-creation with the project team; - Pilot testing supported by process- and effect evaluation (i.e. observations, interviews, food literacy questionnaires).
Output	Intervention function and form	New *intervention form*: UfC online. Adaptations made to intervention materials to ensure fit with the online- and home setting (i.e. home assignments, a closed online notice board, consuming meal separately). Addition of a sixth flexible component—environmental sustainability—in response to new stakeholder collaborations.
	Implementation strategies	Adapted recruitment strategies and CL manual taking into account the online and home setting.
*Phase 4: Scaling up (currently taking place)*		
Input	Context	A regional branch organization took on responsibility for upscaling UfC online, implementation by new professionals (*adopting organizations and stakeholders*). Project team responsible for training, quality assurance and monitoring.
	Stakeholders	- Researchers, PL and CL (project team); - The regional branch of the Dutch initiative *Jong Leren Eten* took the lead in the scale-up of UfC; - Interested adopting organizations targeting similar and new contexts (i.e. childcare); - Diversity of CLs increases (e.g. primary school teachers, dieticians).
	Action-oriented research	- Input of stakeholders through interim bilateral contact or co-creation; - Monitoring reach and implementation (i.e. website data, interviews, registration of implementation).
Output	Intervention function and form	Continuous process of developing new intervention forms.
	Implementation strategies	Support system including an on the spot train-the-trainer for interested CLs was requested.

### Phase 1: Initial intervention development

#### Input: context

Starting point for developing the intervention was the nutrition education intervention Good Affordable Food (GAF) (Bessems *et al.*, 2020), an intervention to promote a healthy diet for adults with a lower SES. Participants and other regional stakeholders involved desired an intervention complementary to GAF, with more focus on practical nutrition knowledge in the form of cooking classes, initiating the development of UfC. The potential reach of groups with a lower SES and effectiveness of such an intervention was considered promising, as other cooking intervention were quite successful in this regard (e.g. Flego *et al.*, 2014; Pooler *et al.*, 2017). Underlying structural factors of SES (i.e. low education level and limited income) were taken into account to match the target groups’ experiences and perceptions (Salmi *et al.*, 2017). A national action programme targeting low literacy funded the initial development.

#### Input: stakeholders

A project team was formed to initiate the development of the intervention, including health promotion researchers, a health promotion professional from the Municipal Health Service, and a cook with experience in nutrition interventions. A supportive stakeholder advisory group was formed to ensure that the intervention aligned with the experience and perceptions of the target group. The stakeholders included librarians, language expertise centers, policy makers and researchers in the field of food literacy. They provided verbal input for and feedback on the blueprints of the intervention and formed a gateway to the target group. In addition, language ambassadors (i.e. adults who had overcome low literacy) and parents (i.e. with lower literacy skills and lower SES) participated in pilot tests and provided feedback on the intervention materials.

#### Input: action-oriented research

A mix of information sources and research methods was used to develop the intervention. The starting point was theoretical and empirical evidence on nutrition interventions specifically targeting parents with lower literacy skills and lower SES (Eldredge *et al.*, 2016; Bartlett, 2017; Bessems *et al.*, 2020; Daalder *et al*., 2021). Intervention materials with similar objectives (e.g. cooking with a limited budget) were reviewed. Interim bilateral contact with professionals in the field added practical experiences related to literacy, social welfare and food literacy. This resulted in an intervention blueprint, which described an initial intervention design (i.e. group size, core components, point of emphasis for flexible assignments), key messages (i.e. small steps to a healthier diet, cooking with a limited budget, practical tips) and implementation strategies (i.e. skills required of the implementer, communication channels).

Early in the development phase, one pilot session was organized on-site with language ambassadors to test the design of the intervention and materials for applicability to literacy. A course leader (CL) implemented the pilot session with the project team and language professionals present. Observations and interviews identified areas for improvement and adjustments to the intervention were discussed with the advisory group. Two pilot tests of four sessions each were then implemented by the CL (September–October 2019) and included a group of parent–child couples, and a group of adults with lower literacy skills. Again, observations and semi-structured interviews with the adopting community centers and CL were used to evaluate target group reach, implementation of the core components, adaptation of flexible components, participant responsiveness and appreciation (e.g. *what was good, what could be improved?*).

Throughout this process, the advisory group provided input on the intervention and implementation strategies in five co-creation meetings. The topics discussed were: (i) the blueprint of the intervention, experiences in reaching adults with lower literacy skills and lower SES (meeting 1); (ii) recruitment strategies, and a first session outline with intervention materials (meeting 2); (iii) output from pilot tests (meetings 3 and 4) and (iv) further regional scale-up (meeting 5). Health promotion researchers chaired the meetings and informed the advisory group about the steps taken. The advisory group was asked to provide feedback on intervention components, share experiences with the target group or similar interventions and discussed it with their constituencies. The meetings were organized between March and November 2019.

#### Output: intervention

Phase 1 developed the core components of the intervention. UfC focussed on taking small steps towards a healthier diet by enabling participants to cook healthy meals. The four food literacy domains—planning, selecting, making (formerly preparing) and eating—were therefore integrated as core components and represented the *intervention function*. More specifically, the intervention focussed on the determinants awareness, attitude, knowledge, skills and self-efficacy (Chapman-Novakofski and Karduck, 2005; Wrieden *et al.*, 2007). The methods and applications used to reach and engage the target group were practical hands-on activities, cooking with an enthusiastic cook, easy recipes and opportunities to taste healthy products (Brown and Hermann, 2005; Condrasky *et al.*, 2006). Furthermore, the advisory group expressed the need to tailor the sessions to the questions, interests and knowledge level of the participants. Making the intervention sensitive to these inputs (i.e. adaptable) was considered a crucial part of the intervention and its function.

Furthermore, the initial *form* of UfC was developed as four weekly or fortnightly sessions of 2.5–3 h for a group of eight adults or parent–child couples. During one session, participants prepared a meal together, under the guidance of a trained CL. All sessions covered activities related to the four literacy domains. Depending on the target group, additional points of emphasis (e.g. healthy eating, literacy skills, saving money on groceries, parenting practices) were captured in flexible assignments (e.g. quiz on food myths, making a grocery list). Assignments and recipes used plain language, step-by-step descriptions and pictures or icons as visual aids.

#### Output: implementation

The implementation strategy was structured around the roles of a project leader (PL) and CL. The PL was responsible for recruiting and supporting new adopters, and promoting the intervention. More specifically, the PL provided advice on recruitment, use of communication channels and integration of the intervention into existing regional projects.

In the early stages of the development, the PL was a joint role of the regional research institution and the regional Municipal Health Service. The CL was responsible for preparing and implementing the sessions. A CL manual that described the structure of the intervention and contained background information on the core and flexible components, supported implementation. A combination of recruitment strategies was used to reach the target group, including face-to-face recruitment where social workers introduced UfC to their clients during regular consultations, social media (video) posts from hosting community centers and several versions of flyers in plain language distributed by community centers or the CL in the neighbouring supermarket. Furthermore, sessions were organized at a familiar location in their neighbourhood, reducing travel time and increasing accessibility.

### Phase 2: Broadening and improving intervention adaptability

#### Input: context and stakeholders

In Phase 2, the project team continued the development process without support from the stakeholder advisory group, as the initial project funding had been exhausted. New adopting organizations and stakeholders emerged (a social non-profit restaurant and a library) who wanted to implement UfC. They aimed to engage elderly people living in poverty (strengthening social cohesion in addition to literacy) and parents with lower literacy skills (improving parenting practices in addition to literacy), respectively. The library also initiated recruitment through primary schools and invited children to actively participate. The wider application of UfC, hence the adaptation process, was initiated. To support this process of adaptation, literature and existing frameworks [e.g. FRAME (Wiltsey Stirman *et al.*, 2019)] were consulted and integrated into the present study.

#### Input: action-oriented research

Mixed research methods were used to closely monitor and support the adaptation process. Intervention-context fit was evaluated through observations and semi-structured interviews with participants, the stakeholders of the non-profit restaurant, the CL, and the PL. Semi-structured interviews were also conducted with the adopting library to evaluate adoption and participant recruitment. Implementation was not evaluated in this case since the actual implementation did not take place due to closure of schools because of the COVID-19 pandemic. To capture adaptations made, observations recorded details regarding implementation (i.e. how was this component implemented, what went differently, list modifications), and interviews included questions about the adaptations needed beforehand to align UfC with other objectives. Again, the results fed the discussion in the project team meetings, in line with the co-creation meetings of Phase 1. Together, this initiated the adjustments to the intervention and implementation strategies.

#### Output: intervention

**Fig. 1:**
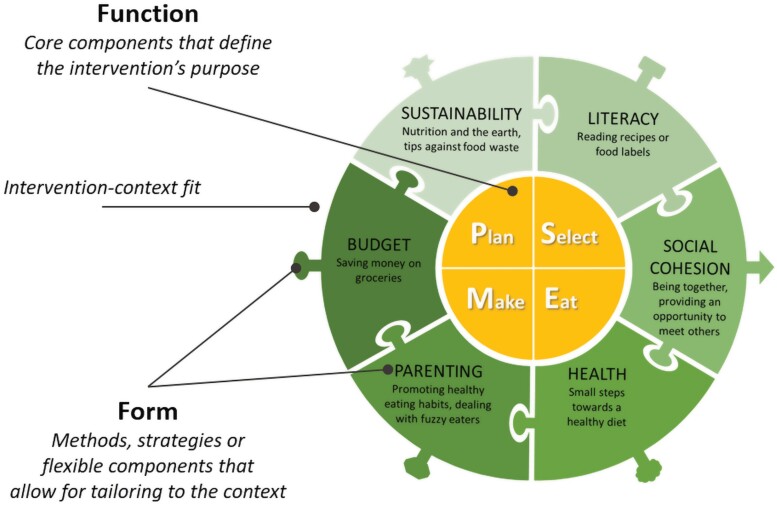
Adaptable intervention UfC as starting point (i.e. unadapted).

Phase 2 resulted in an even broader UfC intervention, by focussing on the interests and needs of the new adopters (e.g. promoting literacy skills, cooking with a limited budget), and on the needs of related target groups (e.g. meeting new people, promoting healthy eating among children). Adapting UfC to new contexts did not seem to result in losing the *function* of the intervention (*yellow core*, Figure 1). Although observations and interviews showed that flexible assignments (e.g. comparing prices of products) were not always or suboptimally implemented, the CL still managed to emphasize the underlying objective (e.g. saving money on groceries) using other methods (e.g. the CL explained how she did budget shopping). This suggested that the intervention form could differ while achieving the same objective (i.e. group discussion on product prices versus completing worksheets on saving money). This resulted in a more comprehensible description of the flexible components (*green adaptable ring*, Figure 1), explaining *why* literacy, social cohesion, health, parenting or budget could be included as a point of emphasis and not *how*. Using this adaptable ring allows the CL to tailor UfC to the context (i.e. target group, adopting stakeholders or organization), resulting in different *intervention forms*. This should ultimately increase intervention-context fit.

#### Output: implementation

With the new description of the intervention, the focus of the implementation process shifted from a traditional protocol to adaptation by the CL to each context. In contrast to a one-size-fits-all intervention, this required a better understanding of the context, the interests of adopting stakeholders and the needs of the target group. It required the CL to have a thorough understanding on how to adapt the intervention to the context. This was clarified in the CL manual with labels and descriptions. Furthermore, a preparation grid was developed for the CLs to explore the objectives of each session (i.e. which core component will predominate, which flexible point of emphasis and associated assignment are important for the target group, which modifications are required to increase fit). Linking flexible assignments was necessary to achieve these objectives and to facilitate both intervention adaptation and implementation. For example, a parent–child group would require other modifications in work formats compared with an adult group. The outline of an example session is included in Supplementary File 1.

### Phase 3: Adaptation to the online setting

#### Input: context and stakeholders

In March 2020, the COVID-19 pandemic led to the first lockdown in the Netherlands. Community centers and primary schools closed and planned UfC sessions on-site were cancelled. To remain applicable as an intervention, an online intervention form was developed to reach families at home in their own kitchens.

The adoption of UfC online by several primary schools sparked the interest of *Jong Leren Eten* (JLE), an initiative of the Dutch ministry of Agriculture, Nature, and Food Quality, and the Ministry of Health, Welfare and Sport, to promote healthy eating at a an early age (Jong Leren Eten, 2022). Again, UfC seemed to align with the objectives of another stakeholder: providing food education. This, together with JLE’s willingness to provide local funding, led to a collaboration from 2021, which led to scaling up and implementation of UfC online (and post-COVID pandemic again on-site) for families through primary schools in the region. In line with JLE’s mission, they also noted that the CL emphasized environmental sustainability during the implementation of UfC. Environmental sustainability was therefore labelled as the sixth flexible component in the existing intervention.

#### Input: action-oriented research

Similar to Phase 1, a combination of methods (i.e. empirical evidence on online cooking interventions, co-creation, pilot testing) fed the adaptations to UfC online. Previous online cooking interventions (Colby *et al.*, 2019; Polak *et al.*, 2019) were used as input to adapt the existing intervention materials, improving their applicability in the online setting. The food literacy domains remained as core components, but were translated to the home setting (i.e. worksheets were adapted into homework assignments). Project team meetings were used to discuss a blueprint of UfC online and to develop a plan for scale-up. One UfC online session was pilot tested in October 2020 to evaluate the online format, applicability of the intervention materials and participant appreciation. Four parent–child couples, with children attending the same primary school, participated in their own home kitchen, via videoconferencing. Observations and semi-structured interviews with the parents and the CL were conducted. Findings were discussed with the project team and the adopting stakeholders (e.g. JLE) to refine UfC online.

A process and effect evaluation was conducted to evaluate the implementation and effectiveness of UfC online. Primary schools adopted UfC online. Process data included semi-structured interviews at multiple time points with a variety of stakeholders (i.e. adopters, participants, the PL and the CL). In addition, observations were used to examine adaptations. Intervention effectiveness was evaluated quantitatively through Food Literacy Behaviour Score Questionnaires adapted from (Begley *et al.*, 2018). Parents completed an online questionnaire 1 week prior to the first session and within 1 week after the last session. These evaluation activities are outside the scope of this article and will be reported elsewhere. The results will be used to inform decisions on national scale-up (Phase 4).

#### Output: intervention

Phase 3 resulted in an additional flexible component in the adaptable ring of UfC (Figure 1), as well as a new *intervention form*: UfC online. Duration of the online session was shortened to 90 min, after which participants consumed the homemade meal with their own families. Flexible assignments were included as homework assignments to be completed outside the session. Before the start of each session, participants received the necessary materials (e.g. recipes, worksheets) and a grocery bag with all the necessary ingredients for one family. Participants cooked live from their own kitchen and interacted with the CL and with each other via video conferencing. The CL guided participants step-by-step through the recipes with verbal and visual explanations, while simultaneously showing how she prepared the meal herself. Everyone at home was welcome to participate, causing a ripple effect to other family members, classmates and neighbours who spontaneously joined the participants. UfC online was supported with child-specific intervention materials, such as a ‘cooking passport’ for weekly notes (i.e. recipes made). For parents, a checklist with preparation steps for setting up the kitchen and instructions for online participation via the videoconferencing software were added to the grocery bag. Finally, a closed online noticeboard was created with all intervention materials and references. This was intended as a long-term reference for families (i.e. sustained implementation at home).

#### Output: implementation

UfC online was adopted by primary schools and community centers and implemented by the CL. Recruitment strategies, materials and implementation strategies were modified to the online setting. For example, flyers were distributed digitally and included instructions on the videoconferencing software. The face-to-face recruitment approach was maintained but was now the responsibility of teachers or other representatives positioned within the implementation location, who were asked to select families with a lower SES, who could benefit from participating in UfC online. At each implementation round, the CL was able to optimize the use of the digital environment in terms of interaction (e.g. use of thumbs up), camera positions and time needed for clear explanations. Paragraphs such as the differences between on-site and online implementation, tasks to consider (e.g. distributing groceries) and technical information (e.g. software tips) were added to the CL manual. The role descriptions of the PL and CL remained the same.

### Phase 4: Scaling up

#### Input: context and supporting stakeholders

Once the function and the different forms of the intervention were established, development entered the scale-up phase. Interest in scaling up increased as the regional stakeholder JLE provided local funding for the dissemination and implementation of UfC among families through primary schools. This caused a role shift with the local funder promoting and supporting the adoption and implementation in new primary schools, as well as recruiting potential new CLs. The project team stepped back and focussed on knowledge sharing (e.g. train-the-trainer), implementation monitoring and quality assurance.

#### Input: action-oriented research

Extensive effect and process evaluations gradually phased out. Monitoring reach and implementation of UfC continued through the intervention website. Interviews with existing stakeholders (i.e. CL, PL and JLE) and feedback on content from interested CLs were used to develop a train-the-trainer module. The scaling up process is currently being monitored through interviews with interested CLs.

#### Output: intervention and implementation

An intervention website describing the intervention and stakeholder experiences was created to disseminate the intervention to other interested stakeholders. In addition, a closed section of the website was used for the free distribution of the CL manual and intervention materials (e.g. flyers, recipes and assignments).

## REFLECTION

This paper demonstrated the use of a non-linear action-oriented approach to develop an adaptable food literacy intervention, increasing intervention-context fit. We derived five important take home messages from our experiences: (i) understand the context; (ii) embrace interdisciplinary collaborations; (iii) learn from your mistakes; (iv) bend, do not break and (v) consider adequate guidance for practice (Box 1). We will reflect and elaborate on each lesson below.

BOX 1: **TAKE HOME MESSAGES**

1. Understand the context you are working in
  Circumstances change. Proverbially, you need to know *which way the wind blows*, in order to navigate, recognise tipping points and follow along if you choose to.
2. Embrace interdisciplinary collaboration
  Stakeholders each have different views on what and how you are trying to achieve your goal. By taking these views into account at every step of the process, you can create a more comprehensive approach, open doors and ultimately increases intervention-context fit.
3. Learn from your mistakes
  Feedback loops allow you to be flexible and adjust as the intervention evolves.
4. Bend, do not break
  As with tree branches and skyscrapers, a ‘flexible’ structure allows for *swaying in the wind* without compromising structural integrity. An adaptable intervention should allow for swaying to prevent core components from breaking.
5. Consider adequate guidance for practice
  Just as participants need support (e.g. personal recruitment, practical tips, lower literacy materials), so do future implementers (e.g. train-the-trainer). Keep an open eye for appropriate implementation strategies and their conditions.

### The need to understand the context you are working with

In line with previous research (Cohen *et al.*, 2008; Chambers and Norton, 2016) we identified the importance of intervention-context fit. We continuously adapted UfC to the needs of new target groups, new stakeholders, new funding opportunities and external circumstances. An action-oriented research approach involving co-creation, pilot testing, observations and interviews was considered to be a valuable approach to gain a comprehensive understanding of the changing context, such as adaptation to UfC online. The pandemic created opportunities for online interventions, as families gained access to online devices through home schooling and working from home and became accustomed to videoconferencing software (Abd-Alrazaq *et al.*, 2021). COVID-19 also called for renewed attention to healthy lifestyles (Van Der Werf *et al.*, 2021). Together, this proved to be a tipping point for UfC to reach families at home. This confirms that adaptable interventions should be seen as an event within a complex system (Hawe *et al.*, 2009; Hawe, 2015). Gathering information about the context in which researchers and implementers work is therefore necessary to fully understand and adequately respond to its complexity (Moore *et al.*, 2021). Co-creation and mixed research methods are appropriate approaches to get closer to the real life setting (Greenhalgh *et al.*, 2016), providing insight into how and why necessary adaptations are made (Holtrop *et al.*, 2022) and enable systematic exploration of these adaptations.

### The need to embrace interdisciplinary collaboration

A variety of stakeholders showed us how the intervention could reach and engage new related target groups with similar challenges and needs. The interest of a growing group of stakeholders and our willingness to adapt UfC to their needs created a snowball effect that resulted in new collaborations within the fields of welfare, education and environmental sustainability, who were willing to fund the local implementation of UfC. It has been argued in the literature that interdisciplinary collaborations reveal important issues that need to be addressed, practice-oriented questions and create intervention receptivity (Oliver *et al.*, 2019). However, we have experienced how this requires cooperativeness and an absence of any hierarchy between stakeholders. Inflexibility, strong personal objectives, lack of necessary skills or financial structures can hinder the process. Instead, a common agenda, open ongoing communication, a trusting relationship and rapid conversion of observations into feedback are required, which is in line with the conditions for Collective Impact (Kania and Kramer, 2011) and other context-oriented research approaches (Bartelink *et al.*, 2023). This implies a role for researchers that goes beyond the research skills of collecting evaluation data, knowledge transfer or facilitating intervention development and implementation. It requires them to be part of the process in practice, but at the same time sufficiently disengaged to be able to oversee and evaluate the bigger picture (Bartelink *et al.*, 2023). In our experience, such extensive process guidance also poses a threat to the objectivity or independence of researchers—a type of tension also identified by Oliver *et al.* (2019). Reporting one’s role as a researcher within an open science approach helps ensure scientific integrity. Furthermore, researchers should be open to requests from the field and weigh input against theoretical and empirical evidence.

### The need to learn from your mistakes

Action-oriented research provided a consistent flow of input and output that provided direct insight into how the intervention was implemented or received in practice. We learned to accept that sometimes a trial and error approach was needed to find out when and why the intervention did not fit the context. For example, we struggled with recruiting participants with lower literacy skills (Phase 1), suboptimal implementation in terms of fidelity (Phase 2) and participant engagement via videoconferencing (Phase 3). The phased non-linear development provided opportunities to learn and time to refine the intervention. Longitudinal evaluations with sufficient time, supported by feedback loops, are therefore important prerequisites for the development of similarly adaptable interventions (Bartelink *et al.*, 2019). Nevertheless, phased development using co-creation is generally a time-consuming process and not feasible for all projects (Greenhalgh *et al.*, 2016). Although this process leads to improved implementation (Bartelink *et al.*, 2019) and is expected to improve sustained uptake (Scheirer and Dearing, 2011; Chambers *et al.*, 2013), sufficient time, funding and flexibility are required.

### The need to build in adaptability from the start

**Fig. 2:**
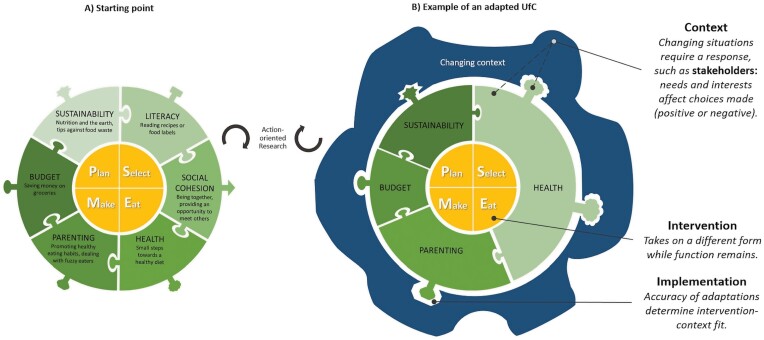
Graphical representation of UfC (A) and an adapted example version with increased intervention-context fit (B).

Intervention adaptability was one of the main building blocks of UfC. Existing frameworks have reported on how to conduct and report adaptations of existing interventions after initial intervention development, while our study incorporates adaptation from the start of the intervention development and elaborately reported this process. The flexible nature (i.e. the combination of core and flexible components) makes UfC an intervention approach rather than a one-size-fits-all intervention (Figure 2). Adaptations are needed to increase intervention-context fit, resulting in a match (Figure 2B). Inadequate adaptations reduce the intervention-context fit and can lead to a mismatch. We have visualized our thought process in Figure 2. The context (*blue ring*) is influenced by the characteristics of the target group, stakeholders and external circumstances (Figure 2B). Examples include objectives for implementing the intervention by adopting stakeholders, or participants’ needs (see Supplementary File 2, for more details). This calls for a response: points of emphasis are selected from the flexible component and tailored to start creating a better *fitting intervention form* (*green adaptable ring*), while the intervention function remains (*yellow core*). The intervention form is complemented by the development of an appropriate implementation strategy (*green puzzle tabs*). The accuracy of the intervention adaptations and the implementation strategy affect the intervention-context fit (*match between tabs and blanks in blue ring*). Within this process, several levels need to be considered. First, a comprehensive view of the target group allows for tailoring and thereby increases intervention-context fit. Second, objectives and interests of stakeholders should be considered in the light of the target group, to avoid conflicting needs. Third, a fitting intervention should be supported by appropriate implementation strategies (e.g. an online intervention to reach families at home). This approach is based on the differentiation between the function and form of the intervention. Intervention developers should be able to identify the function—the core components that play a role in the behaviour change process (Hawe *et al.*, 2004).

To create interventions with build in adaptation (Castro *et al.*, 2004), we advocate for the inclusion of flexible components. The mere presence of such an adaptable ring should initiate the thought process on intervention adaptation and intervention-context fit. We consider this process to be a core step of UfC. This means that from our perspective, the core components and the adaptability together represent *intervention function*. This adds a new dimension to the definition of intervention function. The selection and modification of components in that ring remains flexible and facilitates the development of *intervention forms*. This adaptable structure allows UfC to sway without breaking the core components. Our elaborate report on the development of an adaptable intervention aligns with existing frameworks on adaptation, which is a strength of the present paper. For example, acquiring input of stakeholders as also depicted in IDEA (Miller *et al.*, 2020), differentiating core components and reportation of the adaptations made as recommended in FRAME (Wiltsey Stirman *et al.*, 2019), and the need for implementers to not only navigate the context but also the consequences of such as suggested by MADI (Kirk *et al.*, 2020). Furthermore, Figure 2 builds upon existing guidance for decision-making such as ADAPT (Moore *et al.*, 2021).

### Consider adequate guidance for practice

In contrast to implementation according to a predefined protocol, implementation of an adaptable intervention requires adaptive management skills (Head and Alford, 2015). An implementer should be able to internalize and maintain the function of the intervention, while developing an intervention form that aligns with the target group. In addition, an implementer should be context sensitive to identify and navigate the changing context. Furthermore, scaling up with new implementers is often a complex process (Greenhalgh *et al.*, 2004), and may require more support than a train-the-trainer module. Preparation for scale-up of UfC has yet to be completed. This also means that a number of derivatives of UfC will follow based on different contexts, requiring alternative research methods to determine the impact of each derivative intervention.

### Strengths and limitations

A strength of the present paper is that it is grounded in existing frameworks and research findings (Wiltsey Stirman *et al.*, 2019; Kirk *et al.*, 2020; Miller *et al.*, 2020; Moore *et al.*, 2021). What our development process adds, however, is the consistent input from practice. The action-oriented approach with co-creation and mixed research methods was a strength of the development process, and enabled us to apply our thought process to the local context. An additional strength is the elaborate description of the development process.

Nevertheless, this ongoing process also poses a limitation for our framework as depicted in Figure 2. Currently, the ring of UfC consists of six flexible components, but new feedback may lead to additional components. In line with this, until Phase 3, we worked with one trained CL, who was constantly part of the development and implementation processes and a member of the project team. As a result, discussion of the role of the implementer in the implementation of UfC is limited. As more CLs will be involved in later implementation stages, future research should evaluate related changes on the implementation and adaptation process. Lastly, the present paper aimed to provide insight into the development process and the related lessons learned. Although study results were used to inform this process, they were not reported for feasibility reasons. This could be seen as a limitation of this paper.

## CONCLUSION

With this paper, we provide a practical application of an action-oriented approach developing an adaptable intervention. We hope to advance adaptation research by encouraging others to incorporate adaptability from the start of intervention development. To achieve this, the *intervention function* should be clearly defined and maintained, while *intervention forms* should be used to adapt the intervention to achieve intervention-context fit. Adaptation to changes within the context should be a continuous collaborative process between researchers, practitioners and other stakeholders. This requires a non-linear approach to intervention development. Interdisciplinary collaboration, co-creation, input based on feedback loops and sufficient time are important prerequisites for the development of adaptable interventions.

## Supplementary Material

daad071_suppl_Supplementary_File_S1

daad071_suppl_Supplementary_File_S2

## Data Availability

Data sharing is not applicable to this article as no datasets were generated or analysed during the current study.
