# Supplementary material for: Context matters—the phased development of an adaptable food literacy intervention: Up for Cooking
Source: Health Promot Int. 2023 Jul 14;38(4):daad071. doi: 10.1093/heapro/daad071 (PMC10347970; doi:10.1093/heapro/daad071)
Supplement: daad071_suppl_Supplementary_File_S1 [file daad071_suppl_supplementary_file_s1.docx]

**Supplementary File 1**. **Overview of a session of Up for Cooking**

| **A session can consist of** | **Explanation (*related materials*)** | **Example implementation and adaptations needed for online delivery** | **Food literacy core** | **Flexible component** |
| --- | --- | --- | --- | --- |
| Opening and walk-in | Welcoming participants, creating a sense of community, explaining the setup of the intervention, and enabling participants to ask questions. | On-site: Participants interact with the CL, tries to get to know them better and asks participants about their cooking habits.  Online: Participants, in addition, receive tips on the videoconference software. | *n.a.* | *Social Cohesion* |
| Healthy snacks | Opportunity to discuss, prepare and try healthy products in the form of snacks. Flexibility to link with theory on taste development and repeated exposure.  *Recipes for healthy snacks, flyers of The Netherlands Nutrition Centre.* | On-site: Participants try vegetable sticks with tzatziki dip, prepared by the CL in advance. The CL explains how dip sauces differ in nutritional value and provides practical tips on the healthier choice.  Online: Participants received snack tomatoes in the grocery bag and introduces them by means of a personal anecdote about herself as picky eater. | *Select*  *Make*  *Eat* | *Health*  *Parenting* |
| Introduction to today’s session | Introducing today’s set-up and the recipe(s) (e.g., what, why, how). Flexibility to vary the order in which components are introduced and/or explained in detail.  *Cooking passport, checklist preparation kitchen, instructions videoconference software.* | On-site: Participants receive a brief introduction of the chosen recipe. The CL explains use of seasonal vegetables and the different preparation techniques, after which participants get a task assigned (see below).  Adaptations for online: Content of the grocery bag is explained while the CL holds every item in front of the camera. The CL briefly explains the choice of recipe, explains parent-child tasks, after which participants are responsible for the whole meal preparation at home (see below). | *Plan*  *Select*  *Make* | *Literacy*  *Health*  *Parenting*  *Budget*  *Env. sustainability* |
| Weighing and measuring | Educating participants why and how to use a scale or measuring cup. Flexibility to vary time point in the session and discussion method (e.g., plenary, individually, etc.)  *Measuring cup, flyers or videos on portion sizes.* | On-site: All participants use a scale to weigh ingredients, with guidance of the CL.  Online: All participants received an ‘Eetmaatje’: a measuring cup for rice, pasta, and water combined, including recommended portion size. Participants are asked to use the measuring cup during meal preparation. Afterwards, a short instruction video on the measuring cup is shared. | *Plan*  *Select*  *Make* | *Parenting*  *Budget*  *Env. sustainability* |
| Cooking | Providing participants the experience that preparing a healthy meal can be fun and easy, as well as providing them with the knowledge, skills and tools for it. Flexibility to vary in recipes and point of emphasis (e.g., budget, health).  *Recipes with and without parent-child tasks, video on cutting techniques.* | On-site: Participants prepare a healthy meal together with the CL, who demonstrates cutting techniques, answers questions, interacts with participants and provides tips or feedback.  Online: Participants cooked live from their own kitchen, with step-by-step verbal and visual guidance of the CL who simultaneously prepares the meal herself. The CL monitors progress via videoconference software, asks participants for thumbs up, answers questions and provides practical tips during waiting time. | *Select*  *Make* | *Health*  *Parenting*  *Budget*  *Env. sustainability* |
| Eating, talking, cleaning up | Showing participants the social side of preparing and eating a healthy meal. | On-site: Participants and the CL clear the dining area and kitchen, do the dishes, and divide any left-over food to take home.  Online: not part of the session. | *Eat* | *Social Cohesion*  *Parenting* |
| Flexible assignment ^1^ | Opportunity to educate, discuss, and emphasise flexible components during the session.  Flexibility to vary in discussion methods (e.g., plenary, individually, Q&A, homework, sharing anecdotes). |  | *Plan*  *Select*  *Make*  *Eat* | *Literacy*  *Social Cohesion*  *Health*  *Parenting*  *Budget*  *Env. sustainability* |
|  | 1. **Quiz on healthy eating**   Participants learn how to take small steps towards a healthier diet. | On-site: Participants answer true/false statements in an interactive quiz. Each answer is followed with background information provided by the CL, and experiences from participants.  Online: Participants react to the true/false statements with thumbs up. There is less interaction due to time. | *Plan*  *Select*  *Make*  *Eat* | *Literacy Health* |
|  | 1. **Quiz on healthy eating and children**   Participants learn about the influence of the social environment and their influence on their child(ren). |  | *Plan*  *Select*  *Make*  *Eat* | *Literacy Health*  *Parenting* |
|  | 1. **Making a grocery list**   Participants learn how to plan their food intake and grocery shopping.  Flexibility to emphasise on a specific flexible component. | On-site: Participants are introduced to different methods to make a shopping list (e.g., different apps, pen and paper), after which they practice together with the CL.  Online: Participants receive a homework assignment, which is briefly introduced by the CL. Participants complete the assignment individually. | *Plan*  *Select* | *Health*  *Budget*  *Env. sustainability* |
|  | 1. **Storing food products**   Participants learn about expiration dates, and how to store food products correctly. | On-site: Participants are shown by the CL were to find expiration dates, followed by a discussion on the layout of the refrigerator.  Online: Participants receive a homework assignment which is briefly introduced by the CL. Participants complete the assignment individually. | *Plan*  *Select* | *Health*  *Budget*  *Env. sustainability* |
|  | 1. **Food labels**   Participants learn about nutrition information panels, advertising, and other packaging details. | On-site: Participants listen to an introduction on food labels by the CL. Afterwards, a work-sheet on food labels is distributed.  Online: Participants receive a homework assignment. To introduce the assignment, the CL exemplifies the information with one or two products from the grocery bag. Participants complete the assignment individually. | *Select* | *Literacy*  *Health* |
|  | 1. **Food prices**   Participants learn how to save money on groceries. | On-site: Participants play an interactive group game on product pricing. The CL provides practical tips.  Online: Participants receive a homework assignment which is briefly introduced by the CL. Participants complete the assignment individually. The CL provides tips on how to save money on groceries throughout the session. | *Plan*  *Select* | *Budget* |
|  | 1. **Word search**   Participants practice reading and the use of letters. | On-site: Participants complete a word search.  Online: Not applicable. |  | *Literacy* |
| Closing | Thanking participants for their enthusiasm, creating a sense of community, and enabling participants to verbalise their interests, questions and needs for the upcoming session. |  | *n.a.* | *Social Cohesion* |

^1^To assist implementation, the CL is encourage to complete a preparation grid in which he/she explores the objective of each session (i.e., which core component will predominate at which time point, which flexible component is selected as point of emphasis, etc.)
