# Supplementary material for: Context matters—the phased development of an adaptable food literacy intervention: Up for Cooking
Source: Health Promot Int. 2023 Jul 14;38(4):daad071. doi: 10.1093/heapro/daad071 (PMC10347970; doi:10.1093/heapro/daad071)
Supplement: daad071_suppl_Supplementary_File_S2 [file daad071_suppl_supplementary_file_s2.docx]

**Supplementary File 2: Details on the adapted example version of UfC**

Supplementary Text box 1

| **An example situation to which UfC should be adapted**  A primary school has decided to adopt and implement the UfC intervention. The school is located in a lower SES neighbourhood and the school board aims to offer the intervention to vulnerable families specifically. Healthy eating is their primary objective as this corresponds with the long-term vision of the school and the municipality. To suit the target group, there is a need to take into account parenting practices and budget. Furthermore, a regional stakeholder is willing to fund implementation but requires the intervention to touch upon sustainable food choices. Ideally, all food literacy domains are addressed. However, going through the steps of meal preparation together (make domain) predominates. In this example, the online cooking intervention would be a suitable implementation strategy to empower the families to cook a healthy, affordable meal in their own home environment. Families receive worksheets with practical tips on saving money on groceries, and recipes are vegetarian or based on seasonal products in light of environmental sustainability. |
| --- |

Supplementary Text box 2

| **Levels on which adaptation mismatches can occur**   1. The target group consists of families with a lower SES and would like to discuss saving money on groceries. However, this is neglected or recipes make use of expensive food products. Alternatively, the target group has lower literacy skills and therefore require easy-to-understand materials. However, flyers make use of long and difficult sentences. 2. A regional stakeholder values the use of local, sustainable products during implementation. This requires the CL to develop recipes with expensive food products that are difficult to find, which is not in line with the expectations and experiences of the target group. 3. The decision was made to implement UfC online, whereas the target group did not have the technical skills to use videoconferencing or was unfamiliar with the software used. |
| --- |
